# Cholinergic Interneurons Mediate Fast VGluT3-Dependent Glutamatergic Transmission in the Striatum

**DOI:** 10.1371/journal.pone.0019155

**Published:** 2011-04-22

**Authors:** Michael J. Higley, Aryn H. Gittis, Ian A. Oldenburg, Nina Balthasar, Rebecca P. Seal, Robert H. Edwards, Bradford B. Lowell, Anatol C. Kreitzer, Bernardo L. Sabatini

**Affiliations:** 1 Howard Hughes Medical Institute, Department of Neurobiology, Harvard Medical School, Boston, Massachusetts, United States of America; 2 Division of Endocrinology, Department of Medicine, Beth Israel Deaconess Medical Center, Harvard Medical School, Boston, Massachusetts, United States of America; 3 Departments of Physiology and Neurology, University of California San Francisco School of Medicine, San Francisco, California, United States of America; 4 Departments of Physiology and Neurology, Gladstone Institute of Neurological Disease, University of California San Francisco, San Francisco, California, United States of America; Johns Hopkins, United States of America

## Abstract

The neurotransmitter glutamate is released by excitatory projection neurons throughout the brain. However, non-glutamatergic cells, including cholinergic and monoaminergic neurons, express markers that suggest that they are also capable of vesicular glutamate release. Striatal cholinergic interneurons (CINs) express the Type-3 vesicular glutamate transporter (VGluT3), although whether they form functional glutamatergic synapses is unclear. To examine this possibility, we utilized mice expressing Cre-recombinase under control of the endogenous choline acetyltransferase locus and conditionally expressed light-activated Channelrhodopsin2 in CINs. Optical stimulation evoked action potentials in CINs and produced postsynaptic responses in medium spiny neurons that were blocked by glutamate receptor antagonists. CIN-mediated glutamatergic responses exhibited a large contribution of NMDA-type glutamate receptors, distinguishing them from corticostriatal inputs. CIN-mediated glutamatergic responses were insensitive to antagonists of acetylcholine receptors and were not seen in mice lacking VGluT3. Our results indicate that CINs are capable of mediating fast glutamatergic transmission, suggesting a new role for these cells in regulating striatal activity.

## Introduction

As the primary excitatory neurotransmitter in the central nervous system, the amino acid glutamate mediates synaptic output from the majority of projection neurons in the brain, including pyramidal cells of the neocortex and hippocampus. However, a growing body of evidence indicates that some populations of inhibitory and modulatory neurons may also use glutamate as a co-transmitter. Histological studies have demonstrated glutamate-immunopositive monoaminergic cells in the brainstem and mesencephalon [1] and revealed the presence of phosphate-activated glutaminase, the primary glutamate synthetic enzyme, in cholinergic and dopaminergic neurons [2], [3]. In addition, the Type-2 vesicular glutamate transporter co-localizes with tyrosine hydroxylase in brainstem catecholaminergic neurons [4]. Furthermore, the Type-3 vesicular glutamate transporter (VGluT3) is rarely expressed in traditional glutamatergic neurons but is present in serotonergic cells of the raphe nuclei, in cholinergic neurons of the striatum and basal forebrain, and in GABAergic interneurons of the hippocampus [5], [6], [7], [8]. Ultrastructural analysis has shown that VGluT3 co-localizes with the vesicular acetylcholine (ACh) transporter in presynaptic terminals in the striatum, suggesting a role in neurotransmission [6], [7], [9].

Physiological evidence for synaptic glutamate release from traditionally non-glutamatergic cells was initially limited to cell culture systems in which serotonergic and dopaminergic neurons were shown to make functional glutamatergic autapses [10], [11]. However, recent work has demonstrated that both electrical and Channelrhodospin2 (ChR2)-mediated optical stimulation of midbrain dopaminergic cells can evoke monosynaptic glutamatergic responses in the ventral striatum [12], [13], [14]. Additionally, optical stimulation of serotonergic neurons in the raphe nuclei evokes glutamatergic potentials in hippocampal interneurons capable of triggering action potentials [15].

Striatal cholinergic interneurons (CINs) provide the sole source of ACh in the striatum and are thought to correspond to “tonically active neurons (TANs)” recorded *in vivo* [16], [17]. The activity of TANs is regulated by behavioral context, exhibiting pauses of several hundred milliseconds following presentation of salient sensory stimuli [18], [19]. Although they comprise only ∼3% of all striatal cells, CINs are hypothesized to regulate striatal synaptic transmission and long-term plasticity via nicotinic and muscarinic ACh receptors [20], [21], [22]. However, co-expression of VGluT3 in cholinergic presynaptic terminals suggests that CINs may also utilize glutamate for transmission in some capacity [7], [9]. Recent studies indicate that *in vivo* activity of CINs alters firing of neighboring medium spiny neurons [23], although the possible role of glutamate release in this process was not examined. Previous work suggested that VGluT3 may energetically facilitate loading ACh into vesicles, as VGluT3 knockout mice exhibit reduced evoked cholinergic release [9]. However, it remains unclear whether CIN expression of VGluT3 is sufficient to support functional glutamatergic synapses capable of activating postsynaptic receptors.

To answer this question, we selectively expressed a conditional form of ChR2 in cholinergic neurons of the dorsal striatum, enabling us to reliably evoke action potentials in CINs. Recordings from medium spiny neurons (MSNs) in acute brain slices revealed excitatory postsynaptic potentials (EPSPs) that were triggered by ChR2-mediated firing of CINs and were blocked by glutamate receptor antagonists. The fractional contribution of NMDA-type glutamate receptors (NMDARs) were significantly different for CIN-evoked glutamatergic responses compared to those arising from optical stimulation of corticostriatal projections, suggesting differential activation of postsynaptic receptors by these two afferent populations. Similar CIN-evoked glutamatergic EPSPs were seen in MSNs using a second, independent mouse line that expresses Cre recombinase in cholinergic neurons but could not be elicited in mice lacking VGluT3. Thus, our results demonstrate that direct and VGluT3-dependent release of glutamate from striatal CINs can evoke fast glutamatergic responses in postsynaptic targets, suggesting a previously unknown role for these interneurons in the control of striatal circuitry.

## Results

**Figure 1:**
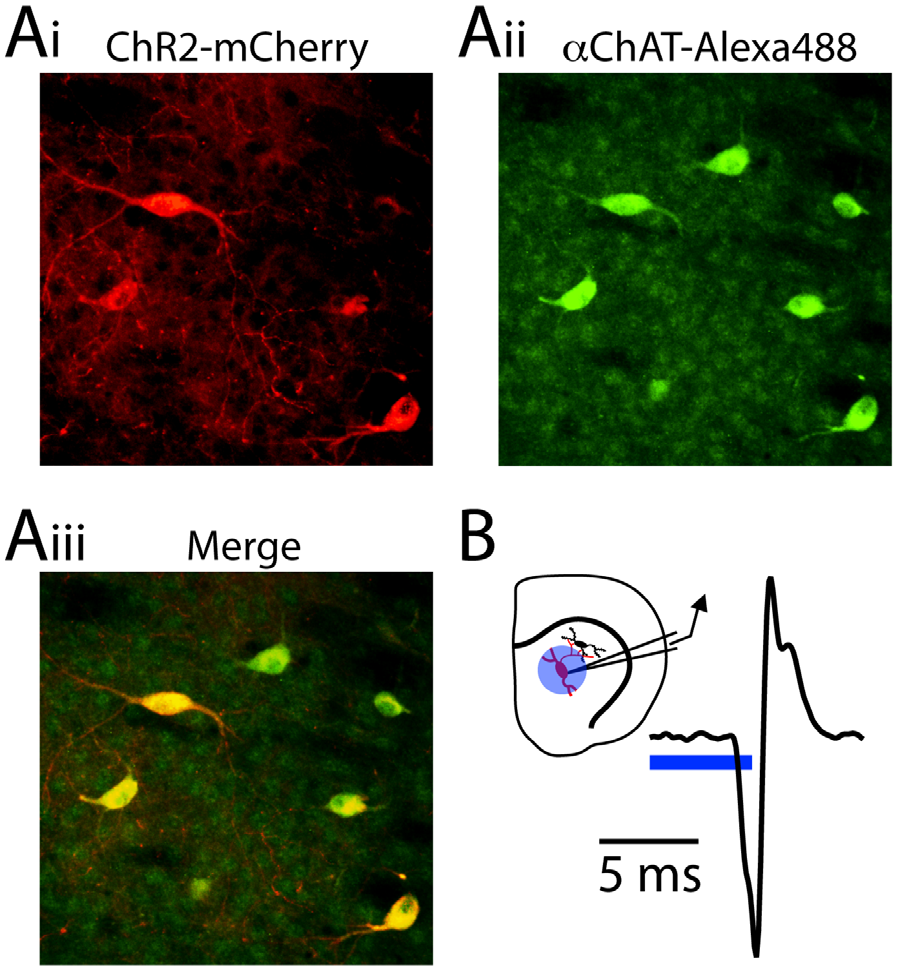
ChR2-mediated activation of striatal cholinergic interneurons. (Ai) Confocal image of mCherry-positive ChR2-expressing neurons in the dorsal striatum of a ChAT-IRES-Cre mouse injected with AAV encoding DFI-ChR2-mCherry. (Aii) Fluorescence immunohistochemical staining for ChAT reveals cholinergic neurons. (Aiii) Merged image. (B) *inset,* Schematic diagram of the recording conditions: cell-attached recordings were made from ChR2-expressing CINs (red) in the dorsal striatum and blue light was delivered to the surrounding area (blue circle) through the microscope objective. *main panel,* Example action potential recorded in cell-attached mode from a ChR2-expressing CIN evoked by a 4 ms pulse of 473 nm light.

To selectively activate striatal cholinergic interneurons (CINs), we utilized knock-in mice that express Cre-recombinase downstream of the native choline-acetyltransferase promoter and an internal ribosomal entry site (IRES) [24]. ChAT-IRES-Cre mice express Cre-recombinase in cholinergic neurons of the striatum, basal and septal nuclei, and neocortex (Rossi et al., 2011). Intracranial injection of adeno-associated virus encoding a Cre-dependent (double floxed inverted) Channelrhodopsin2-mCherry fusion protein (DFI-ChR2-mCherry) [25], [26] into the striatum of these mice enabled the CIN-specific expression of the light-activated excitatory ChR2, which was clearly observed 7–14 days after injection (Fig. 1A, see methods).

We characterized the ability of ChR2 to drive CIN activity in an acute brain slice using cell-attached recordings obtained from ChR2-positive CINs that were visually identified under epifluorescence (Fig. 1B). Recordings were made at room temperature to reduce spontaneous firing of CINs [17]. Brief pulses (2–5 ms) of whole-field blue light delivered through the microscope objective reliably evoked action potentials (APs) in CINs with a mean latency measured from the start of the light pulse of 4.2±0.5 ms (n = 8), demonstrating that virus mediated Cre-conditional expression of ChR2 provides a viable tool for driving activity in this population of genetically-defined local interneurons.

**Figure 2:**
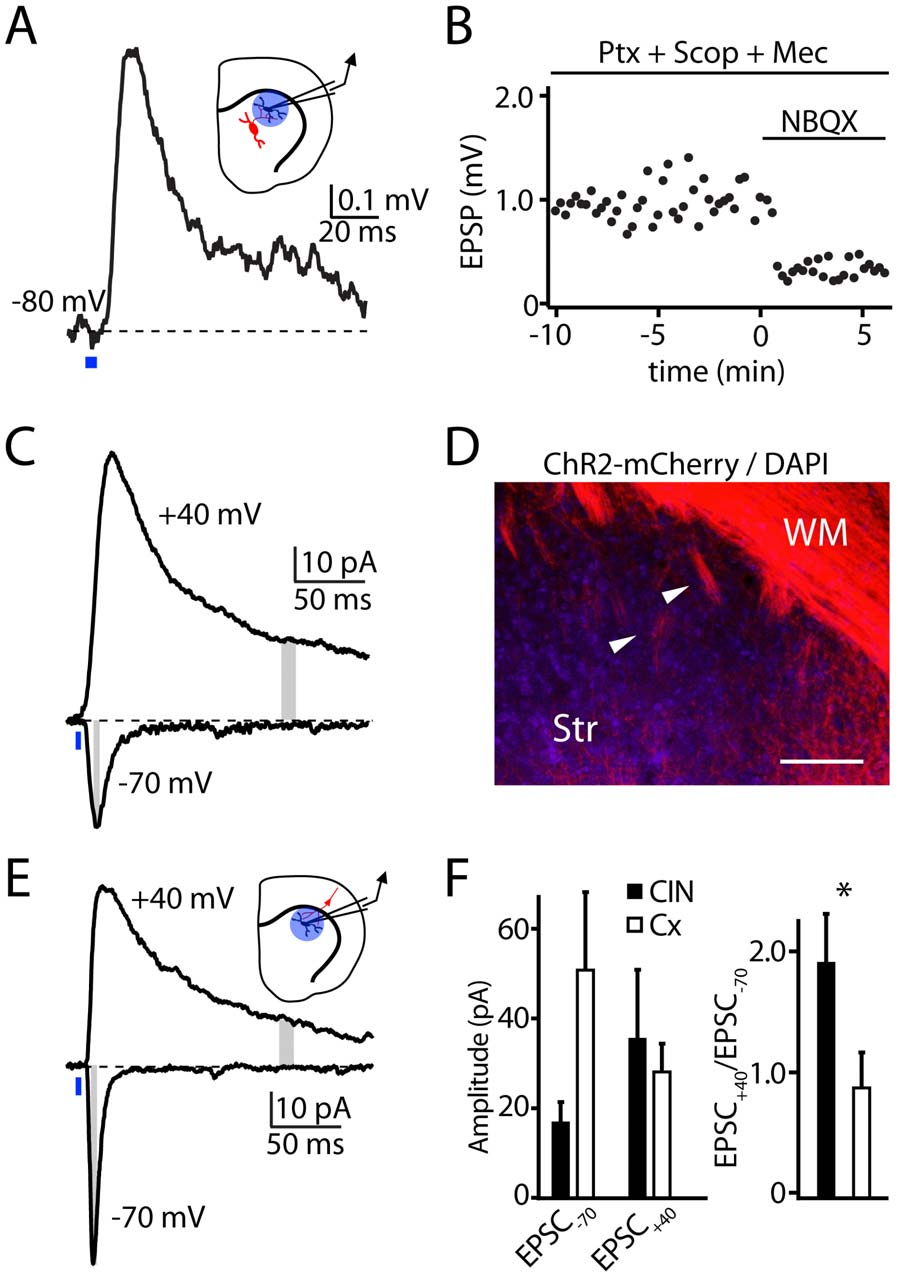
Light-evoked CIN action potentials evoke glutamatergic responses in MSNs. (A) *inset,* Schematic diagram of the recording conditions: whole-cell recordings were made from MSNs that neighbored ChR2-expressing CINs (red). The blue circle indicates the region stimulated by blue light. *main panel,* Example EPSP recorded in an MSN in response to a 4 ms light pulse (blue bar). (B) Amplitudes of light-evoked EPSPs in the presence of antagonists of GABA_A_ (Picrotoxin, Ptx), muscarinic (Scopolamine, Scop), and nicotinic (Mecamylamine, Mec) receptors and following application of the AMPA/kainate glutamate receptor antagonist NBQX. (C) Example of light evoked (blue bar) CIN-mediated EPSCs in a voltage-clamped MSN at holding potentials of −70 and +40 mV demonstrating the large current that is visible more than 100 ms after the light pulse at +40 mV. The amplitudes of the rapid −70 and prolonged +40 mV EPSC components were measured in the periods indicated by the gray bars. (D) Confocal image of mCherry-positive ChR2-expressing fibers in the motor cortex, white matter (WM) and underlying striatum (Str). Large bundles of corticofugal fibers (white arrowheads) and diffuse small axonal collaterals are visible throughout the dorsolateral striatum. Scale bar 200 µm. (E) *inset,* Schematic diagram of the recording conditions: whole-cell recordings were made from MSNs neighboring ChR2-expressing corticostriatal fibers (red), and blue light was delivered to the region indicated by the blue circle. *main panel,* Example EPSCs recorded in an MSN in response to a blue light pulse at the indicated holding potentials. (F) *left*, Average amplitudes of light-evoked EPSCs measured in MSNs held at either −70 or +40 mV in response to ChR2-mediated activation of either CINs or corticostriatal fibers (Cx). *right*, Average ratio of +40/−70 mV current amplitudes measured following ChR2-mediated activation of either CINs or corticostriatal fibers.

Previous studies of CIN function in the striatum have focused on the actions of nicotinic and muscarinic ACh receptors, which modulate glutamate, GABA, and dopamine release at striatal synapses and directly depolarize the principal striatal cells, medium spiny neurons [MSNs, 20,21,22]. To determine whether activation of CINs could produce a direct postsynaptic response, we made current-clamp recordings from MSNs in brain slices containing CINs expressing ChR2 (Fig. 2). Pulses of blue light applied through the microscope objective to the area surrounding the recorded MSN evoked a transient depolarization from a resting potential of −80 mV (Fig. 2A). On average (n = 10), the evoked response had an amplitude of 1.9±0.5 mV and occurred with a latency of 7.0±0.5 ms from the light pulse onset.

The light-evoked response was resistant to blockade of GABA_A_ receptors (50 µM picrotoxin) as well as nicotinic and muscarinic ACh receptors (1 µM mecamylamine and 10 µM scopolamine, respectively), indicating that it was not mediated by acetylcholine receptor-dependent direct depolarization or by indirect reversed inhibition via activation of nicotinic receptor-expressing GABAergic interneurons (Fig. 2B). Instead, the depolarization was sensitive to application of the AMPA/kainate-type glutamate receptor (AMPAR) antagonist NBQX (10 µM), which significantly reduced the response magnitude to 22.4 ± 2.5% of control (n = 5, Student's T-test p<0.001, Fig. 2B). The remaining response was eliminated by the NMDA-type glutamate receptor (NMDAR) antagonist CPP (10 µM, data not shown). Thus, our results indicate that firing of CINs generates glutamatergic excitatory postsynaptic potentials (EPSPs) in nearby MSNs.

As CIN-evoked EPSPs exhibited a NBQX-resistant component, we further examined the relative contribution of NMDA-type glutamate receptors (NMDARs) to CIN-evoked currents. Voltage-clamp recordings were obtained from MSNs at room temperature using a cesium-based internal solution, in the prensence of picrotoxin, mecamylamine, and scopolamine. Light-evoked excitatory postsynaptic currents (EPSCs) were measured at hyperpolarized and depolarized membrane potentials. At a holding potential of −70 mV, CIN stimulation evoked an average (n = 7) inward peak current of 16.7±4.4 pA (Fig. 2C), estimating the contribution of AMPARs. It is possible that these measurements are contaminated with a contribution of current flow through NMDARs, although this is likely due to the Mg block of NMDARs and the lack of a prolonged phase of the EPSC at this potential. In the same cells, we quantified the NMDAR contribution as the current evoked at a holding potential of +40 mV, measured 150 ms after the light pulse (35.2±15.5 pA). The average ratio (+40:−70) of these measures was 1.9 ± 0.4. (Fig. 2F).

To directly compare optically-evoked glutamatergic inputs from CINs with similarly evoked inputs from cortical afferents, we expressed a Cre recombinase-independent version of the ChR2-mCherry fusion protein in motor cortex neurons projecting to the striatum [27]. At 7–14 days following injection, axonal fibers descending from the overlying cortex could clearly be seen branching throughout the dorsal striatum (Fig. 2D). As above, we made voltage-clamp recordings in MSNs and delivered light pulses through the microscope objective (Fig. 2E), evoking EPSCs with an average (n = 5) latency to 4.1±0.3 ms. From a holding potential of -70 mV, the average peak inward current amplitude was 50.5±17.3 pA, while from a holding potential of +40 mV, the average current at 150 ms was 28.2±6.4 pA (Fig. 2E,F). Calculating the ratio of currents at the two holding potentials (+40:−70) yielded an average value of 0.86 ± 0.3, significantly smaller than for currents evoked by stimulation of CINs (p<0.05, Student's T-test, n = 5, Fig. 2F). These experiments, performed at room temperature, indicate that ChR2-mediated activation of striatal CINs from a quiescent state is capable of evoking glutamatergic responses in striatal MSNs that is independent of cholinergic actions.

**Figure 3:**
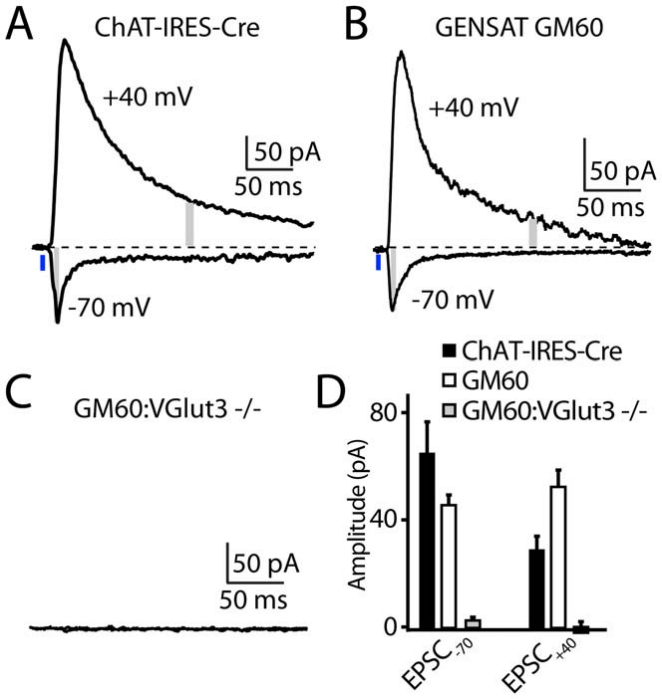
CIN-mediated glutamatergic currents in MSNs require VGluT3 expression. (A) Example light-evoked EPSCs at 32–34°C in an MSN held at −70 or +40 mV in an acute striatal slice of a DFI-ChR2-mCherry AAV injected ChAT-IRES-Cre mouse. (B) As in Panel A, showing recordings at 32–34°C obtained from an MSN in an acute slice of a DFI-ChR2-mCherry AAV injected GM60 mouse that expresses Cre under control of a BAC spanning the ChAT genomic locus. (C) As in Panel B, showing failures to evoke EPSCs in an MSN of a DFI-ChR2-mCherry AAV injected GM60; VGluT3^−/−^ mouse. (D) Average light-evoked EPSC amplitudes measured in MSNs at holding potentials of −70 and +40 mV in acute slices prepared from mice of the indicated genotypes.

In order to confirm both the robustness of these findings and that they represent vesicular release of glutamate by CINs, several additional experiments were performed (Fig. 3). First, analysis of ChR2-mediated glutamatergic responses in MSNs was repeated at near physiological temperatures (32–34°C) at which CINs are spontaneously active (mean firing frequency in cell-attached recordings  = 1.6±0.3 Hz, n = 6). At this temperature, brief blue light illumination also triggered EPSCs in MSNs at holding potentials of −70 mV (116.4±18.7 pA) and +40 mV (29.7±5.4 pA, Fig. 3A, D). Importantly, the smaller relative NMDAR contribution, in comparison to data in Figure 2, was due to the faster kinetics of the evoked EPSC at near-physiological temperatures which results in substantial decay by 150 ms after stimulus.

Second, in order to confirm the robustness of our findings in an independent mouse line, similar experiments were performed in the GENSAT GM60 mouse line in which Cre is expressed in cholinergic interneurons following integration of a bacterial artificial chromosome spanning the ChAT genetic locus and in which the ChAT coding sequence has been replaced with that of Cre [28]. As above, blue light stimulation of acute slices prepared from DFI-ChR2-mCherry AAV-infected mice elicited EPSCs in MSNs at both −70 and +40 mV (Fig. 3B). On average (n = 7), these currents were 46.7±3.6 pA and 52.8±5.9 pA, for −70mV and +40mV, respectively, Fig. 3D.

Third, although the lack of sensitivity of the observed responses in MSNs to cholinergic and GABAergic antagonists suggests direct release of glutamate from CINs, we examined the dependence of CIN-mediated glutamatergic EPSCs in MSNs on the expression of the vesicular glutamate transporter VGluT3. Importantly, in the striatum, this transporter is expressed exclusively in CINs. GM60 mice were bred with VGluT3^−/−^ mice [29] to generate GM60;VGluT3^−/−^ animals. Blue-light stimulation of DFI-ChR2 AAV infected striatal slices of these mice failed to trigger EPSCs at holding potentials of −70 or +40 mV (n = 6, Fig. 3C,D). Thus, glutamatergic EPSCs in MSNs triggered by optogenetic stimulation of ChR2-expressing CINs requires VGluT3, strongly suggesting that VGluT3 expression in CINs is required to package glutamate within vesicles that are subsequently released in an activity-dependent manner.

## Discussion

Here, we demonstrate that action potentials in striatal CINs generate glutamatergic postsynaptic responses in MSNs that exhibit distinct properties compared to activation of cortical afferents. The ability of CINs to mediate glutamatergic transmission is consistent with reports that these cells express the vesicular glutamate transporter VGluT3, which co-localizes with the vesicular ACh transporter in the same presynaptic terminals [6], [7], [9]. Earlier work suggested that the function of VGluT3 at these synapses was to facilitate cholinergic vesicle loading, as the ACh transporter requires anion co-entry for continual activity [9]. Moreover, transgenic mice lacking VGluT3 show reduced ACh release in striatal slices [9]. Our results show that, in addition to supporting cholinergic signaling, VGluT3 expression is necessary for CIN-mediated glutamatergic responses in MSNs. However, our results do not allow us to determine whether individual vesicles contain both ACh and glutamate.

As CIN “function” has generally been assessed using bath-applied cholinergic pharmacological agents, our findings highlight the importance of studying the endogenous synaptic outputs of these cells. Along with recent studies demonstrating glutamatergic release from dopaminergic and serotonergic cells [12], [13], [14], [15], our results suggest that glutamate transporter expression may be a general mechanism used in modulatory neurons to increase their repertoire of neurotransmitters. In addition, the reliable relationship between a single presynaptic action potential and a single postsynaptic EPSP at short latency (∼3 ms) strongly suggests that CINs release glutamatergic vesicles via conventional exocytosis machinery. These results are supported by the finding that CIN-evoked glutamatergic currents are not seen in MSNs of mice lacking VGluT3, the sole vesicular glutamate transporter expressed in CINs.

We found that EPSCs evoked by CIN stimulation exhibited a significantly larger fractional contribution of NMDARs when compared to those triggered by stimulation of cortical afferents. Ultrastructural analyses indicate that cholinergic terminals are typically apposed to dendritic shafts and the necks of dendritic spines, in contrast to the spine heads targeted by cortical synapses [30]. Thus, one possible explanation for the large NMDAR contribution is that glutamate from CINs activates a distinct set of postsynaptic receptors. Alternatively, glutamate from CINs may diffuse from the release site to nearby postsynaptic densities under traditional synaptic contacts. The relatively higher glutamate affinity of NMDARs versus AMPARs [31] might explain the greater NMDAR activation by this volumetric signal. This model is consistent with the hypothesis that ACh acts primarily via volume transmission [32].

Our results also indicate that, in the context of ongoing spontaneous (>1 Hz) activity, the addition of a single ChR2-triggered spike is able to trigger a postsynaptic response, suggesting that glutamate release is maintained at these firing rates. Future studies are necessary to determine if similar release also occurs at higher rates *in vivo*, and in animals outside of the 3–4 week old age group studied here.

The function of glutamate release by CINs is unclear. One possibility is that VGluT3 is necessary for effective vesicular loading of ACh at CIN terminals, and glutamate release is an unintended byproduct of the subsequent co-localization of these two transmitters. Alternatively, the tonic activity of CINs *in vivo* [19] may provide a steady release of low levels of glutamate into the striatum. Synaptic plasticity in the striatum is thought to be critical for normal motor learning, and many forms are dependent on activation of both NMDARs and metabotropic glutamate receptors [33]. Thus, tonic CIN activity may keep plasticity systems in a constant low level of activation. Conversely, *in vivo* TANs, which are thought to correspond to cholinergic neurons, transiently reduce their firing in response to behaviorally relevant sensory cues [19], suggesting that appropriately timed pauses in glutamate release may contribute to learning stimulus-reward associations.

## Methods

### Slice preparation and pharmacology

All animal handling was performed in accordance with institutional and federal guidelines guidelines and were approved by the Harvard (03551) and UCSF (AN083939-01) Institutional Animal Care and Use Committees. These protocols include specific approval for the animal procedures used in this study – i.e. stereotactic intracranial virus injection and euthanasia followed by tissue harvest. Recordings were made from CINs or MSNs in striatal slices taken from male and female postnatal day 22–28 mice that were euthanized under isoflurane anesthesia. Coronal slices (300 µm thick) were cut in ice-cold external solution containing 110 mM choline, 25 mM NaHCO_3_, 1.25 mM NaH_2_PO_4_, 2.5 mM KCl, 7 mM MgCl_2_, 0.5 mM CaCl_2_, 25 mM glucose, 11.6 mM sodium ascorbate, and 3.1 mM sodium pyruvate, bubbled with 95% O_2_ and 5% CO_2_. Slices were then transferred to ACSF containing 127 mM NaCl, 25 mM NaHCO_3_, 1.25 mM NaH_2_PO_4_, 2.5 mM KCl, 1 mM MgCl_2_, 2 mM CaCl_2_, and 25 mM glucose, bubbled with 95% O_2_ and 5% CO_2_. After an incubation period of 30–40 minutes at 34°C, the slices were maintained at 22–25°C until use. Experiments were conducted at room (22–24°C) or near-physiological (32–34°C) temperatures as noted in the text. For some experiments (see text), one or more of the following drugs (Tocris) were added to the ACSF at the following concentrations (in µM): 50 picrotoxin, 10 scopolamine, 1 mecamylamine, 10 NBQX, and 10 CPP.

In order to address concerns that the presence of choline in the cutting solution might have altered the physiology of CINs, experiments were repeated using slices that were cut either in ice-cold standard ACSF or in a sucrose-based cutting solution consisting of (in mM): 79 NaCl, 23 NaHCO3, 68 sucrose, 12 glucose, 2.3 KCl, 1.1 NaH2PO4, 6 MgCl2, and 0.5 CaCl2. No differences were noted across these conditions, and results were pooled for presentation.

### Electrophysiology and imaging

Cell-attached recordings (voltage-clamp mode, holding potential adjusted to eliminate any holding current) were obtained from red-fluorescing (ChR2-mCherry-positive) CINs identified with video-IR/DIC and epifluorescence. Whole-cell recordings were obtained from MSNs identified with video-IR/DIC. For current-clamp recordings, glass electrodes (2–3.5 MΩ) were filled with internal solution containing (in mM): 135 KMeSO_3_, 10 HEPES, 4 MgCl_2_, 4 Na_2_ATP, 0.4 NaGTP, and 10 Na_2_CreatinePO_4_, adjusted to pH 7.3 with KOH. For voltage-clamp recordings, cesium was substituted for potassium. Recordings were made using an Axopatch 200B amplifier, and data was filtered at 5 kHz and digitized at 10 kHz. Data in Fig. 3B,C were collected with a Molecular Devices Multiclamp 700B. Liquid junction potentials were not corrected and estimated as ∼8 mV.

### Viral Channelrhodopsin2 expression and activation

To express the light-activated membrane channel Channelrhodopsin2 (ChR2) in either CINs or corticostriatal afferents, we injected 1.0 µl of a recombinant adenoassociated virus (serotype 2/8, Harvard Gene Therapy Institute) into the striatum or motor cortex of p8–p14 mice, respectively. The virus carried either a double-floxed-inverted Channelrhodopsin2-mCherry construct under control of the Ef1a promoter (CINs) or non-conditional construct coding for Channelrhodopsin2-mCherry under the synapsin promoter (corticostriatal) [34]. After 7–14 days post-injection, mice were euthanized for slice preparation as above and exhibited strong labeling of targeted cells. Selective expression of ChR2 in CINs was verified by staining striatal slices from injected mice with a primary antibody raised against ChAT (Millipore AB144P, 1∶1000). To activate Channelrhodopsin2-positive fibers, light from a 473 nm laser (Optoengine) was focused onto the back aperture of the microscope objective, producing a wide-field exposure around the recorded cell of 10–20 mW/mm^2^. Brief (2–5 ms) pulses of light were delivered under control of the acquisition software. In some experiments, blue light was restricted to a ∼10×300 micron stripe in the field of view using a cylindrical lens. Results obtained with this illumination arrangement were not significantly different than those obtained with whole-field illumination.

### Data acquisition and analysis

Data were acquired using National Instruments and Heka ITC-18 D/A data acquisition boards and custom software written in MATLAB (Mathworks). Off-line analysis was performed using custom routines written in MATLAB and Igor Pro (Wavemetrics). The amplitudes of all EPSPs and non-NMDAR-mediated EPSCs were calculated by averaging over a 3 ms window around the peak, whereas a 10 ms window was used to calculate amplitudes of NMDAR-mediated EPSCs at 150 ms following the stimulus.
